# Nuclear galectin-1-FOXP3 interaction dampens the tumor-suppressive properties of FOXP3 in breast cancer

**DOI:** 10.1038/s41419-018-0448-6

**Published:** 2018-03-16

**Authors:** Yuan Gao, Xiaoju Li, Zhen Shu, Kuo Zhang, Xiaochang Xue, Weina Li, Qiang Hao, Zhaowei Wang, Wangqian Zhang, Shuning Wang, Cheng Zeng, Dong Fan, Wei Zhang, Yingqi Zhang, Huadong Zhao, Meng Li, Cun Zhang

**Affiliations:** 1 State Key Laboratory of Cancer Biology, Biotechnology Center, School of Pharmacy, The Fourth Military Medical University, 710032 Xi’an, People’s Republic of China; 2 Institute of Material, Medical School of Pharmacy, The Fourth Military Medical University, 710032 Xi’an, People’s Republic of China; 3 Department of General Surgery, Tangdu Hospital, The Fourth Military Medical University, 710038 Xi’an, People’s Republic of China

## Abstract

FOXP3 is an important X-linked suppressor of breast cancer. It is reported that FOXP3 is usually mutant, absent, or cytoplasmic distribution in breast cancer cells, which increases the risk of breast cancer. However, in our study the full-length FOXP3 transcript can be detected in breast cancer cells and nuclear FOXP3 is expressed in some breast cancer samples. Therefore, an important question is how the tumor-suppressive function of wild-type FOXP3 is negated in these cancers. We found that Gal-1 is a novel interacting protein of FOXP3 in breast cancer. Furthermore, our results show that the FKH domain in FOXP3 is essential for its interaction with Gal-1. Through ChIP-seq assay, we found that the expression of Gal-1 could inhibit a variety of target genes which were directly regulated by FOXP3. More importantly, these FOXP3-bound genes are involved in the development and metastasis of cancer. Furthermore, functional studies revealed that blocking the FOXP3/Gal-1 interaction restores the tumor-suppressive properties of FOXP3 in breast cancer cells. Finally, we observed that the nuclear abundance of Gal-1 was significantly higher in breast cancer tissues than that in adjacent normal tissues. In addition, we identified that the acidic extracellular microenvironment in breast cancer tissues causes Gal-1 to accumulate in the nucleus. Altogether, nuclear Gal-1 interferes with the binding of FOXP3 to DNA by interacting with the FKH domain of FOXP3, and it indicates a possible mechanism for the loss of the tumor-suppressive properties of FOXP3 in wild-type FOXP3-positive breast cancer.

## Introduction

The transcription factor FOXP3 is a member of the FOX protein family, which contains a characteristic DNA-binding forkhead (FKH) domain^1^. FOXP3 functions as the master regulator of Tregs^2^. Recently, FOXP3 expression in different tumor cells has been found. Although it is reported that FOXP3 can promote tumor growth in melanomas^3^ and that FOXP3 blockade improves the therapeutic efficacy by inhibition of Tregs and through a direct anti-tumor effect in breast cancer^4^, extensive studies suggest a tumor suppressor role for FOXP3 in breast cancer^5,6^. The suppression of FOXP3 expression can induce dysregulation of many oncogenes, such as *MYC*, *SKP2*, and *ERBB2*, which are involved in the progression of breast cancer^6–9^. In addition, FOXP3 is reported to suppress breast tumor progression, by physically interacting with Runx1^10^. FOXP3 can suppress tumor growth, by regulating the expression of miR-146a/b^11^. Moreover, FOXP3 plays an important role in breast cancer metastasis, by regulating the expression of CXCR4 and SATB1^12,13^. Given the importance of FOXP3 as a breast cancer suppressor, it is surprising that a substantial number of breast cancer samples are FOXP3-positive^5,14^. Therefore, an important question is how the tumor-suppressive function of FOXP3 is negated during the development of breast cancer.

As a member of the β-galactoside-binding protein family, Galectin-1 (Gal-1) is composed of 135 amino acids and encoded by the *LGALS1* gene, which contains four exons^15^. Gal-1 contains a single carbohydrate-recognition domain by which it can bind the N-acetyllactosamine (LacNAc) epitopes present in extracellular glycans, such as lactose^16^. Although Gal-1 lacks a signal peptide, it is found in the extracellular matrix of various normal and neoplastic tissues^17^. Outside the cell, Gal-1 can mediate cell–cell and cell–ECM contacts by interacting with glycoproteins, such as laminin and fibronectin. For example, the metastatic spread of cancer cells occurs partially through the interaction of Gal-1 and glycoproteins in the extracellular matrix^18^. In addition, within the cell, Gal-1 is found in the cytosol and nucleus. Even though some studies have reported that intracellular Gal-1 plays roles in signal transduction and transcription in a carbohydrate-independent manner^18,19^, the role of intracellular Gal-1, especially nuclear Gal-1, remains to be elucidated.

Here, we demonstrate the presence of the full-length FOXP3 transcript in some breast-cancer tissues. Our results indicate a novel function for nuclear Gal-1, in mediating the loss of the tumor-suppressive function of FOXP3 through interaction with the FKH domain and inhibition of the DNA-binding ability of FOXP3 in breast cancer cells.

## Results

### The expression of FOXP3 is detected in breast cancer tissues

The full-length FOXP3, consisting of 431 amino acids, is expressed within the nucleus of normal epithelial cells^6,20^. To assess the expression of FOXP3 in breast cancer cells, we analyzed single-cell RNA-sequencing data on primary breast cancer cells (GSE75688). We found that the full-length FOXP3 transcript was detected in 6 breast cancer cases (6/11, 54.5%) (Supplementary Figure 1a). Furthermore, we examined FOXP3 expression, in human primary breast cancer tissues, from 165 breast cancer patients. This analysis demonstrated that nuclear FOXP3 was expressed in 32.1% of breast cancer samples (Supplementary Figure 1b–c, Supplementary Table 1). This suggests that although FOXP3 is usually absent in breast cancer, there are a substantial number of breast cancer samples that are positive for FOXP3.

### Gal-1 interacts with FOXP3

**Fig. 1:**
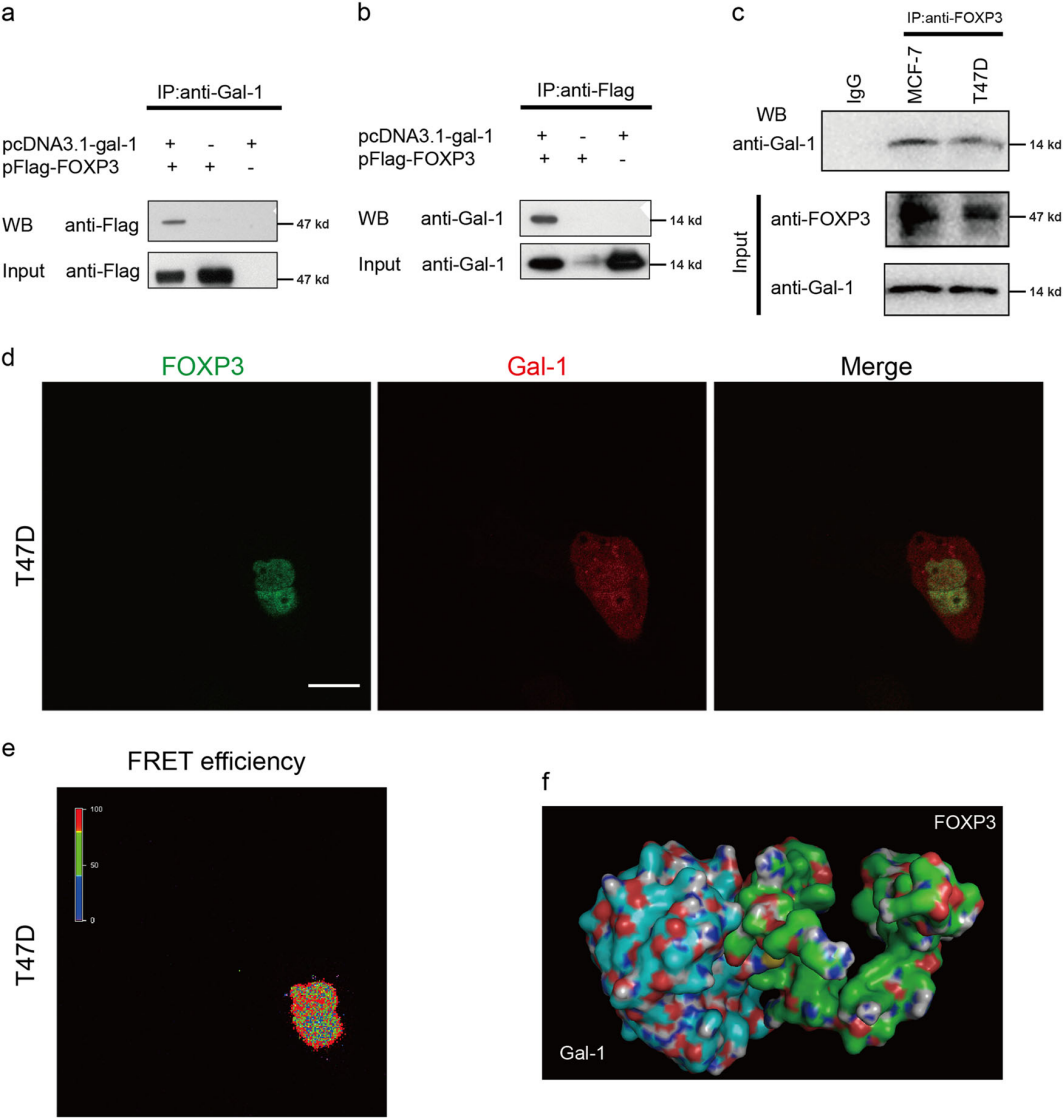
Gal-1 interacts with FOXP3. Exogenous FOXP3 interacts with Gal-1. HEK293T cells were transfected with Flag-tagged FOXP3 (or empty vector) and Gal-1 (or empty vector). Co-IP experiment was performed using (**a**) anti-Gal-1 antibody or (**b**) anti-Flag antibody. The immunoprecipitates were analyzed via western blotting with anti-Flag or anti-Gal-1 antibody. **c** Endogenous FOXP3 interacts with Gal-1. Co-IP experiment was performed using anti-FOXP3 antibody in MCF-7 and T47D cells. IgG was used as the negative control. The immunoprecipitates were analyzed via western blotting with anti-FOXP3 and anti-Gal-1 antibodies. **d** T47D cells were transfected with FOXP3-eGFP and Gal-1-dsRed. FRET microscopy was used to observe the localization of the fusion proteins in these cells. Scale bar = 20 μm. **e** Cells in **d** were analyzed for FRET signals. The color bar represents FRET efficiency (purple indicates the absence of FRET signal). **f** Visualization of the FOXP3/Gal-1 complex. The overall structure is shown as a cartoon, where each protein is colored differently. FOXP3 is shown in green, and Gal-1 is shown in blue

To investigate the mechanisms by which the tumor-suppressive function of FOXP3 is inhibited, we focused on the interaction of FOXP3 with other proteins. Using the yeast two-hybrid assay, we screened for proteins that can interact with FOXP3 (Supplementary Figure 2a). The potential FOXP3-interacting proteins are listed in Supplementary Table 2. Among these, Gal-1, a molecule known to be upregulated in invasive breast cancer^21,22^ and reported to play crucial roles in tumor metastasis, proliferation, and angiogenesis^23^, drew our attention. To confirm whether FOXP3 can interact with Gal-1, we co-transfected Flag-tagged FOXP3 and Gal-1 into HEK293T cells and performed co-IP assay. After immunoprecipitation with anti-Gal-1 antibody, Flag-FOXP3 was detected in the precipitates derived from the cells transfected with both Flag-tagged FOXP3 and Gal-1 (Fig. 1a). Reciprocally, after immunoprecipitation with anti-Flag antibody, Gal-1 was detected in the co-immunoprecipitation complex, derived from the cells transfected with both Flag-tagged FOXP3 and Gal-1 (Fig. 1b). Then, we performed a co-IP assay to test the interaction between endogenous FOXP3 and Gal-1. After immunoprecipitation with anti-FOXP3 antibody, we observed that there was a significant staining of Gal-1 in the samples (MCF-7 and T47D cells) (Fig. 1c). These indicated that FOXP3 could interact with Gal-1 in breast cancer cells. Additionally, the FOXP3-eGFP and Gal-1-dsRed fusion proteins were constructed and co-transfected into MDA-MB-231 cells. We observed that the majority of cells showed nuclear colocalization of FOXP3-eGFP and Gal-1-dsRed (Supplementary Figure 2b–c). Meanwhile, the immunofluorescence assay showed that native Gal-1 expression was found both in the cytoplasm and nucleus of MDA-MB-231 cells, and that few native FOXP3 expression was found in MDA-MB-231 cells (Supplementary Figure 2d). More importantly, to investigate whether Gal-1 could interact with FOXP3 in situ (live cells), we performed fluorescence resonance energy transfer (FRET) analysis. FRET is a powerful technique that monitors the energy transfer between donor (GFP) and acceptor (RFP) fluorescent proteins, and it can be used to measure distances between 1 and 10 nm, which can be further used to characterize the proximity of interacting molecules^24^. The FOXP3-eGFP and Gal-1-dsRed fusion proteins were co-transfected into T47D cells. As shown in Fig. 1d, FOXP3-eGFP was mainly distributed in the nucleus, and Gal-1-dsRed was expressed in both the cytoplasm and nucleus. Furthermore, based on the results shown in Fig. 1d, positive FRET signals were detected between FOXP3-eGFP and Gal-1-dsRed in T47D cells, and found to be limited to the nucleus (Fig. 1e). Therefore, the results of FRET analysis suggest that the interaction between Gal-1 and FOXP3 mainly occurs in the nucleus. In addition, a model of the FOXP3/Gal-1 complex was generated via computational docking, and this model also revealed that Gal-1 was a potential interacting protein of FOXP3 (Fig. 1f).

### The FKH domain of FOXP3 is essential for its interaction with Gal-1

**Fig. 2:**
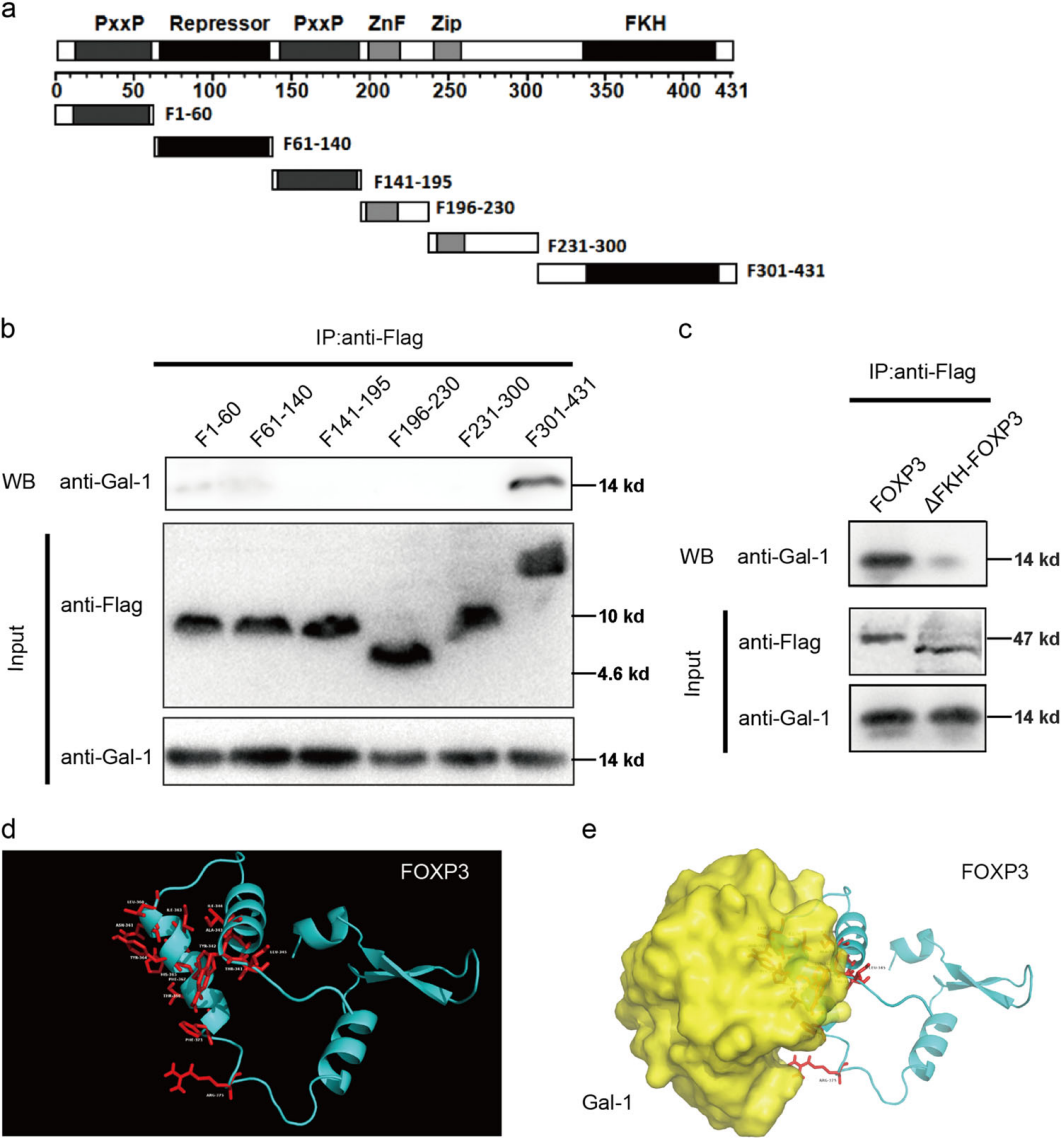
The FKH domain of FOXP3 is essential for its interaction with Gal-1. **a** Schematic representation of full-length FOXP3 and corresponding truncated FOXP3 proteins (called F1-60, F61-140, F141-195, F196-230, F231-300, or F301-431). **b** HEK293T cells were transfected with corresponding Flag-tagged truncated FOXP3 and Gal-1. Co-IP experiment was performed using anti-Flag antibody. **c** HEK293T cells were transfected with Flag-tagged FOXP3 or Flag-tagged ΔFKH-FOXP3 and Gal-1. Co-IP experiment was performed using anti-Flag antibody. **d** Three-dimensional structure of FOXP3. The key residues of the FKH domain are shown in red. **e** A computational docking model shows the superimposition of Gal-1 (yellow) and FOXP3

To further investigate the mechanism of interaction between FOXP3 and Gal-1, a series of FOXP3-deletion mutants were constructed (Fig. 2a). We co-transfected the corresponding Flag-tagged truncated FOXP3 and Gal-1 proteins into HEK293T cells and performed co-IP assay. The results showed that the F301-431 variant, which retained the FKH domain, could bind to Gal-1 (Fig. 2b). These data indicated that the FKH domain of FOXP3 is essential for its interaction with Gal-1. Furthermore, a FKH-deleted FOXP3 mutant was constructed and cloned into the pFLAG vector (Flag-tagged ΔFKH-FOXP3). We co-transfected Flag-tagged FOXP3 or Flag-tagged ΔFKH-FOXP3 and Gal-1 into HEK293T cells and performed co-IP assay. The results showed that the deletion of the FKH domain from FOXP3 dramatically abolished the interaction of FOXP3 with Gal-1 (Fig. 2c). In addition, the precise three-dimensional (3D) structure of FOXP3, from the above FOXP3/Gal-1 complex, suggested that the FKH domain of FOXP3 was occupied by Gal-1 (Fig. 2d–e). Taken together, these results not only confirmed that the DNA-binding FKH domain of FOXP3 is required for binding Gal-1, but also suggested that the DNA-binding ability of FOXP3 might be abolished by its interaction with Gal-1 in the breast cancer cells.

### The N-terminus of Gal-1 is critical for interaction with FOXP3

**Fig. 3:**
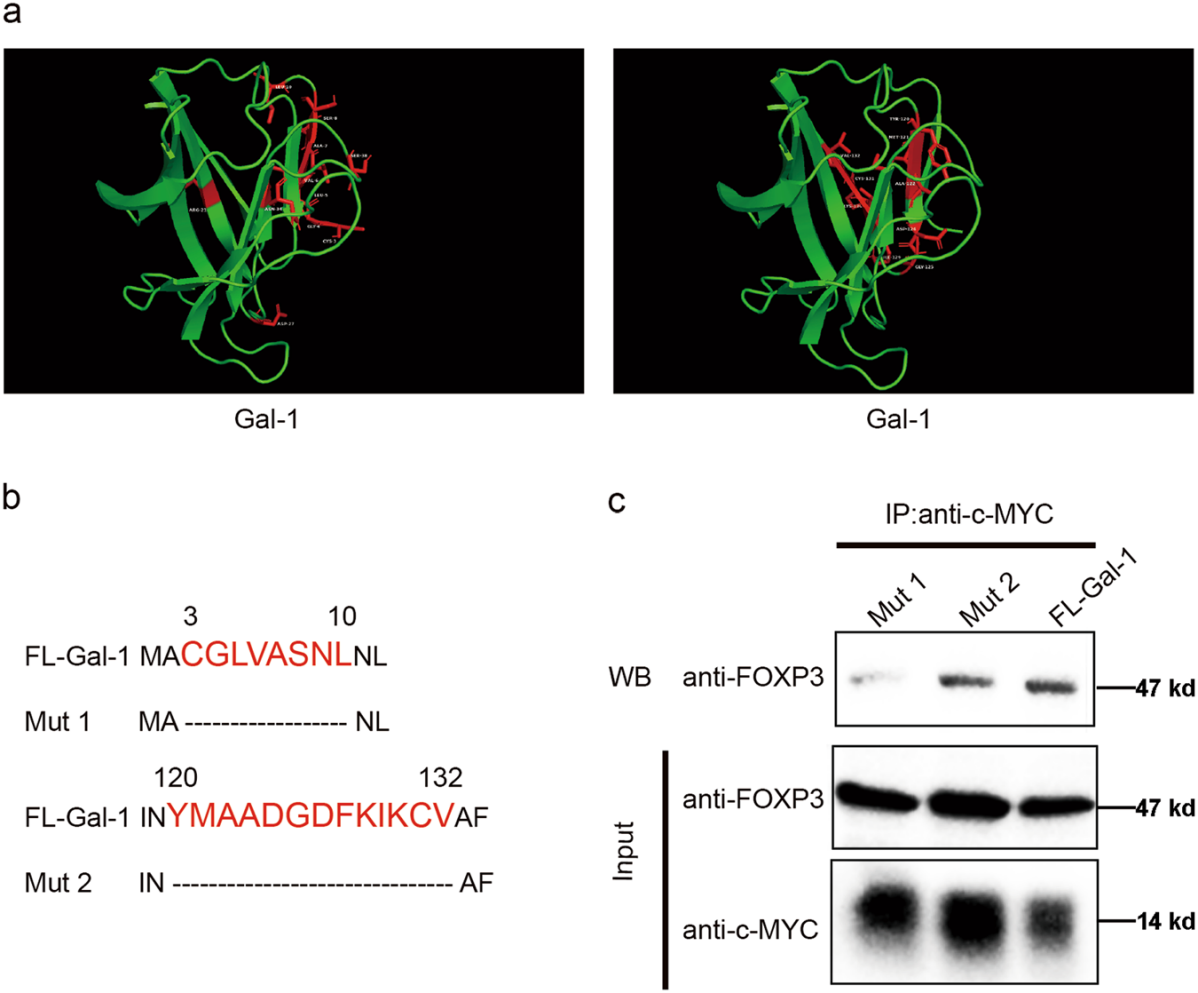
The N-terminus of Gal-1 is critical for interaction with FOXP3. **a** Three-dimensional structure of Gal-1. The key residues, which are potential FOXP3 binding sites, are shown in red. **b** Schematic representation of two potential Gal-1-FOXP3 interaction sites in Gal-1 (indicated in red). FL-Gal-1 full-length Gal-1, Mut1 deletion of CGLVASNL amino acid sequence, and Mut2 deletion of YMAADGDFKIKCV amino acid sequence. **c** HEK293T cells were transfected with c-MYC-tagged Mut1, Mut2, or Gal-1 and FOXP3. Co-IP experiment was performed using anti-c-MYC antibody

We further identified the regions of Gal-1 responsible for FOXP3–Gal-1 interaction, using the detailed 3D structure of Gal-1 from the above-mentioned FOXP3/Gal-1 complex, and found two potential FOXP3 binding sites on Gal-1 (Fig. 3a). Therefore, the indicated regions were deleted (Fig. 3b). Given that the deletion of indicated regions might affect the recognization of anti-Gal-1 antibody to the corresponding Gal-1 mutants, we used vectors encoding MYC-tagged Gal-1 or MYC-tagged deletion mutants (Mut1, Mut2) in our study. In the co-IP assay, we used anti-MYC antibody to co-precipitate the complex. The results from co-IP assay showed that the deletion of the third to the tenth amino acids in the N-terminus of Gal-1 (Mut1) could dramatically abolish the interaction of FOXP3 and Gal-1, suggesting that this region is important for the binding between Gal-1 and FOXP3 (Fig. 3c).

### The expression of Gal-1 alters the genome-wide binding patterns of FOXP3

**Fig. 4:**
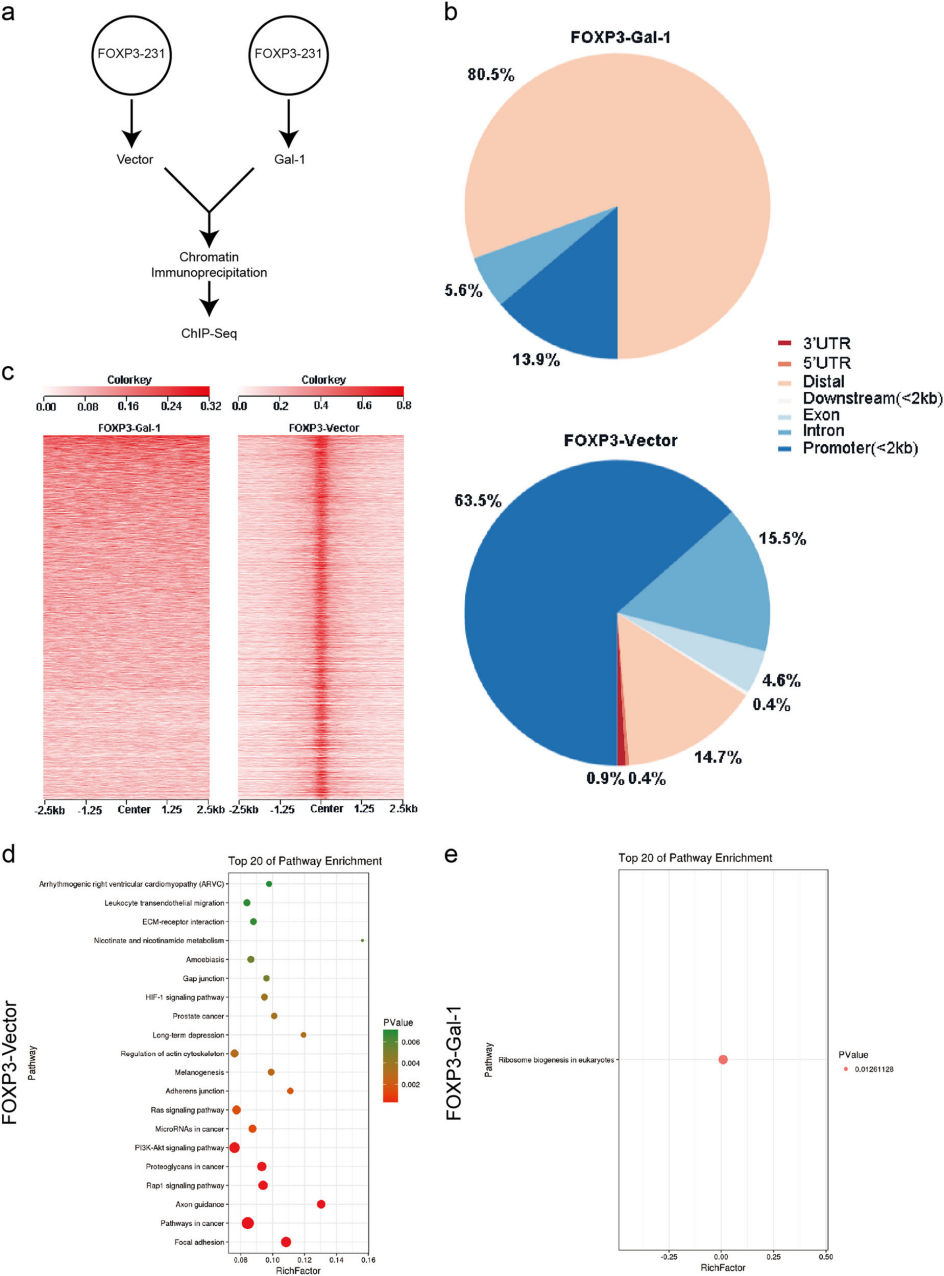
The expression of Gal-1 alters the genome-wide binding pattern of FOXP3. **a** Schematic overview of the breast cancer cell sample preparation for ChIP-Seq analysis. FOXP3-MDA-MB-231 cells were transfected with Gal-1 or empty vector before chromatin immunoprecipitation. **b** Genomic distributions of FOXP3 peaks in FOXP3-MDA-MB-231 cells transfected with Gal-1 or empty vector. **c** Heat maps of FOXP3 binding with FOXP3-MDA-MB-231 cells transfected with Gal-1 or empty vector. Each line on the *y*-axis represents a genomic region flanking the FOXP3 peak. **d** KEGG analysis of FOXP3-bound genes in FOXP3-MDA-MB-231 cells transfected with empty vector. **e** KEGG analysis of FOXP3-bound genes in FOXP3-MDA-MB-231 cells transfected with Gal-1

It has been reported that the FKH domain of FOXP3 is the main region responsible for its binding to the DNA and the genetic influence^25^. To determine whether the nuclear expression of Gal-1 affects the binding of FOXP3 to DNA throughout the genome, a genome-wide ChIP-Seq analysis was performed^26^. Thus, MDA-MB-231 cells, stably expressing FOXP3 (FOXP3-MDA-MB-231), were derived. After the endogenous Gal-1 was knockdown to eliminate the background effect, the FOXP3-MDA-MB-231 cells were transfected with Gal-1 (FOXP3-Gal-1) or empty vector (FOXP3-vector) and then subjected to ChIP-Seq analysis (Fig. 4a). We analyzed the numbers of chromatin regions that were enriched for FOXP3 from each group of cells. In cells transfected with Gal-1, the overall recruitment of FOXP3 to promoter regions was lower than that in the cells transfected with empty vector (13.9% vs. 63.5%) (Fig. 4b). The heat maps of FOXP3-binding signals in the FOXP3-MDA-MB-231 cells transfected with Gal-1 or empty vector are shown in Fig. 4c. GO analysis revealed that the genes exhibiting differential FOXP3 recruitment can be categorized into biological processes, including cell morphogenesis, locomotion, cytoskeleton organization, growth, cell junction organization, cell cycle, cell motility, cell death, and cell proliferation (Supplementary Figure 2e). More importantly, KEGG analysis revealed that the enriched signal pathways, related to the target genes of FOXP3, are mainly involved in cancer-related pathways, and these were almost abolished by co-transfection with Gal-1 (Fig. 4d–e). SATB1, Bcl-2, and TGF-β1 were in the differentially FOXP3-bound genes, and FOXP3 is associated with the expression of IL-10 and TGF-β1^3^. Therefore, to verify the result of the ChIP-Seq analysis, SATB1, Bcl-2, TGF-β1, and IL-10 were chosen and identified by real-time PCR assay. We found that SATB1, Bcl-2, and TGF-β1 were upregulated in FOXP3-Gal-1 cells, compared to FOXP3-vector cells. No statistically significant difference was found in IL-10 expression between the two groups (Supplementary Figure 3a–d). Furthermore, the supernatants were collected for the analysis of cytokine production, (TGF-β1) by ELISA. We found that FOXP3 could inhibit the production of TGF-β1, while Gal-1 could affect this FOXP3 mediated event (Supplementary Figure 3e). Collectively, these results suggest that the expression of Gal-1 in FOXP3-positive breast cancer cells may dampen the tumor-suppressive properties of FOXP3 by interacting with the FKH domain of FOXP3.

### The effect of Gal-1-FOXP3 interaction on the tumor-suppressive properties of FOXP3

**Fig. 5:**
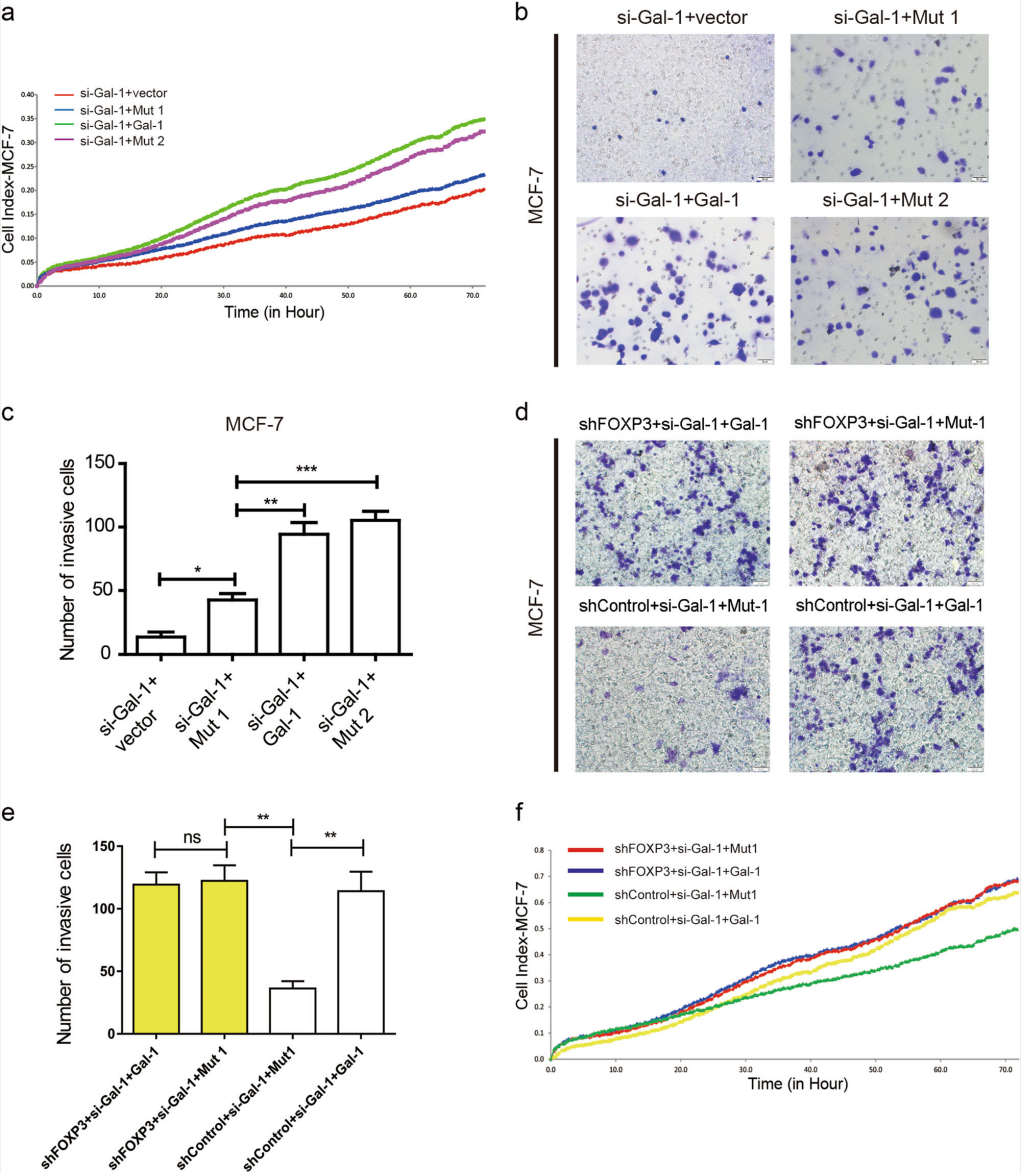
The effect of FOXP3/Gal-1 interaction on the tumor-suppressive function of FOXP3. **a** siRNAs specifically targeting Gal-1 were transfected into MCF-7 cells to knockdown endogenous Gal-1, then these cells were transfected with vectors expressing Mut1, Mut2, or Gal-1 or empty vector, and subjected to xCELLigence RTCA. **b** A transwell assay was performed to determine the invasive capability of si-Gal-1-transduced MCF-7 cells transfected with vectors expressing Mut1, Mut2, or Gal-1, or empty vector. Scale bar = 50 μm. **c** Quantification of invasive cells from **b** (*n* = 3). siRNA specifically targeting Gal-1, was transfected into shControl-MCF-7 or shFOXP3-MCF-7 cells to knockdown endogenous Gal-1, then these cells were transfected with vectors expressing Mut1, or Gal-1, and subjected to (**d–e**) the transwell assay or (**f**) xCELLigence RTCA. Scale bar = 50 μm. **e** Quantification of invasive cells from **d** (*n* = 3). **c,e** The data are shown as the mean ± s.e.m. ns, *P* > 0.05; *P < 0.05; **P < 0.01; and ***P < 0.001 (**c**) ANOVA with Dunnett’s *t* test. **e** ANOVA with Tukey *t*-test

Due to its suppressive effect on the proliferation and metastasis of cancer cells, FOXP3 is considered a suppressor of breast cancer^11–13^. Therefore, to evaluate the biological effect of the FOXP3/Gal-1 interaction on the tumor-suppressive properties of FOXP3, we performed the xCELLigence RTCA^27,28^ to observe the proliferation of breast cancer cells. siRNAs specifically targeting Gal-1 were transfected into MCF-7 cells (that express endogenous FOXP3) or FOXP3-MDA-MB-231 cells to knockdown endogenous Gal-1. si-Gal-1#1 was chosen for subsequent experiments, based on its interference efficiency (Supplementary Figure 4a–d, Supplementary Table 3). After the knockdown of endogenous Gal-1, MCF-7 cells were transfected with vectors expressing Mut1, Mut2, or Gal-1 or empty vector, and subjected to xCELLigence RTCA. The results showed that the cells expressing Mut2 or Gal-1 grew significantly faster than the cells expressing Mut1 (Fig. 5a) (Supplementary Figure 5a). A similar phenomenon was observed in the FOXP3-MDA-MB-231 cells (Supplementary Figure 5b-c). Moreover, transwell assays were performed using endogenous Gal-1-knockdown MCF-7 and FOXP3-MDA-MB-231 cells, transfected with vectors expressing Mut1, Mut2, or Gal-1 or empty vector. The results showed that, compared with that of the Mut2 or Gal-1 group, the number of invasive cells was lower in the Mut1 group (Fig. 5b–c, Supplementary Figure 5d–e).

Then we silenced endogenous FOXP3 in MCF-7 cells by shRNA (Supplementary Figure 5f). After the knockdown of endogenous Gal-1, shControl-MCF-7, or shFOXP3-MCF-7 cells were transfected with vectors expressing Mut1 or Gal-1. These cells were subjected to transwell and xCELLigence RTCA assays. We found that, Mut1 transfected shControl-MCF-7 cells (expressing endogenous FOXP3) showed lower proliferative and invasive ability than Gal-1 transfected shControl-MCF-7 cells. However, no statistically significant difference was found in proliferative and invasive capacity between Gal-1 transfected shFOXP3-MCF-7 cells and Mut1 transfected shFOXP3-MCF-7 cells. (Fig. 5d–f) (Supplementary Figure 5g). These results indicated that the low proliferative and invasive capacity of Mut1-transfected shControl-MCF-7 cells is mediated by the release of FOXP3 function.

### Breast cancer tissues express higher levels of nuclear Gal-1 than corresponding non-cancerous tissues

**Fig. 6:**
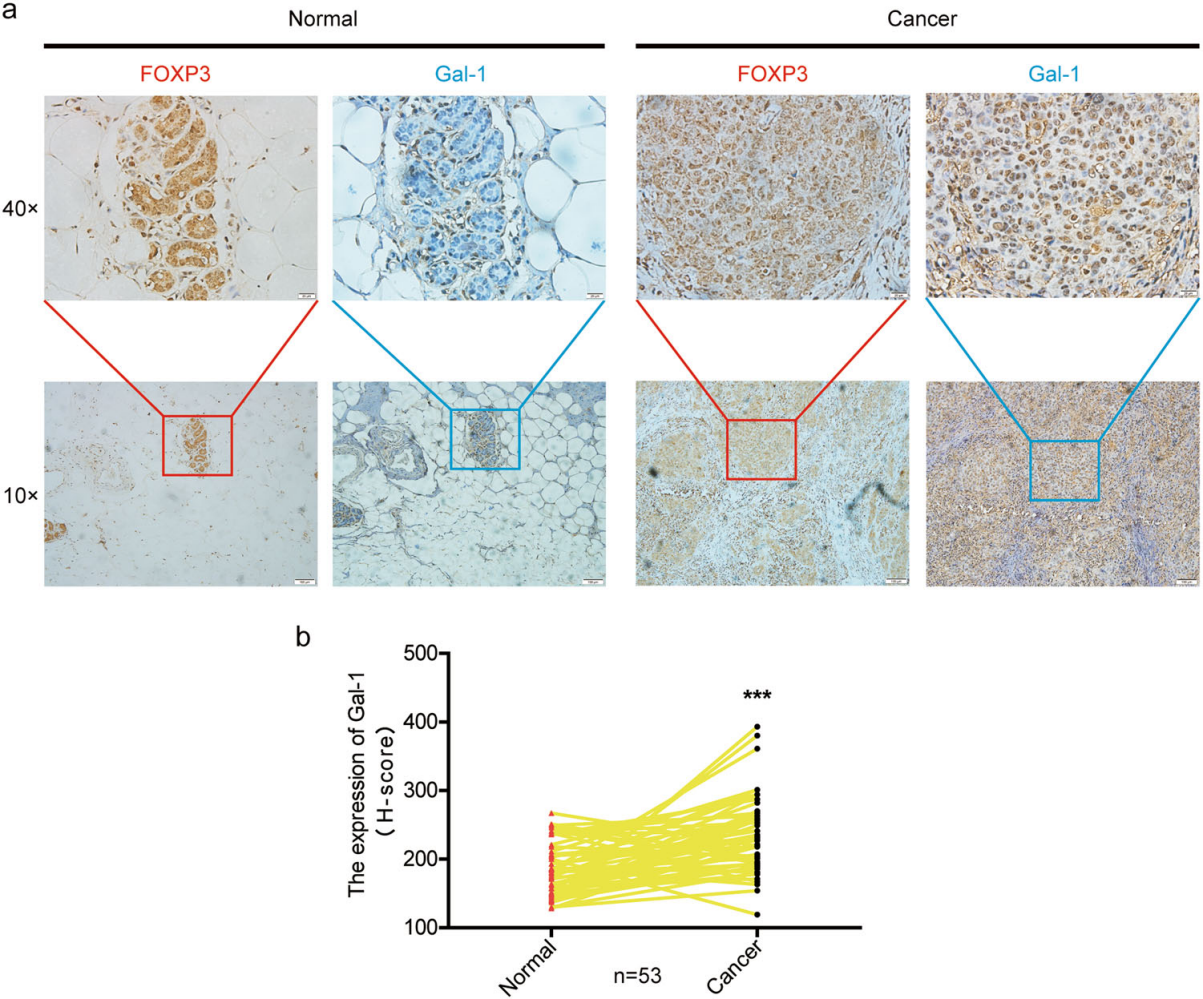
Breast cancer tissues express higher level of nuclear Gal-1 than corresponding non-cancerous tissues. Serial sections were used for the immunohistochemical staining of FOXP3 and Gal-1. **a** Representative immunohistochemical staining for FOXP3 and Gal-1 in normal tissues and primary tumor tissues from breast cancer patient. Scale bars = 100 μm (×10) and 20 μm (×40). **b** The nuclear Gal-1 expression score was quantified and analyzed in the normal tissues and primary human breast cancer tissues. ***P < 0.001. **b** Paired *t*-test

As a transcription factor, FOXP3 can exert its tumor-suppressive activities in the nucleus, by regulating a network of oncogenes involved in various cellular functions. The above results suggested that the differential expression of Gal-1 in breast cancer and adjacent normal tissue samples may lead to the loss of the tumor-suppressive function of FOXP3. To test this hypothesis, 53 paired nuclear FOXP3-positive breast cancer tissue and adjacent normal tissue, among those shown in Supplementary Table 1, were used to measure the expression of nuclear Gal-1. As shown in representative images (nuclear FOXP3 expressed in both breast cancer tissue and adjacent normal sample), the abundance of nuclear Gal-1 was higher in primary breast cancer tissue than in adjacent normal tissues (Fig. 6a) (Supplementary Figure 6a). In addition, by analyzing the 53 cases of nuclear FOXP3-positive breast cancer samples, we found that there was no statistically significant difference between the expression of nuclear Gal-1 and breast carcinoma characteristics, such as age, clinical stages, ER status, and PR status (Supplementary Table 4). Consistent with this, the level of nuclear Gal-1 was significantly higher in nuclear FOXP3-positive breast cancer tissues, compared with adjacent normal tissues in the 53 paired samples (Fig. 6b).

### Extracellular lactose controls the nuclear localization of Gal-1

**Fig. 7:**
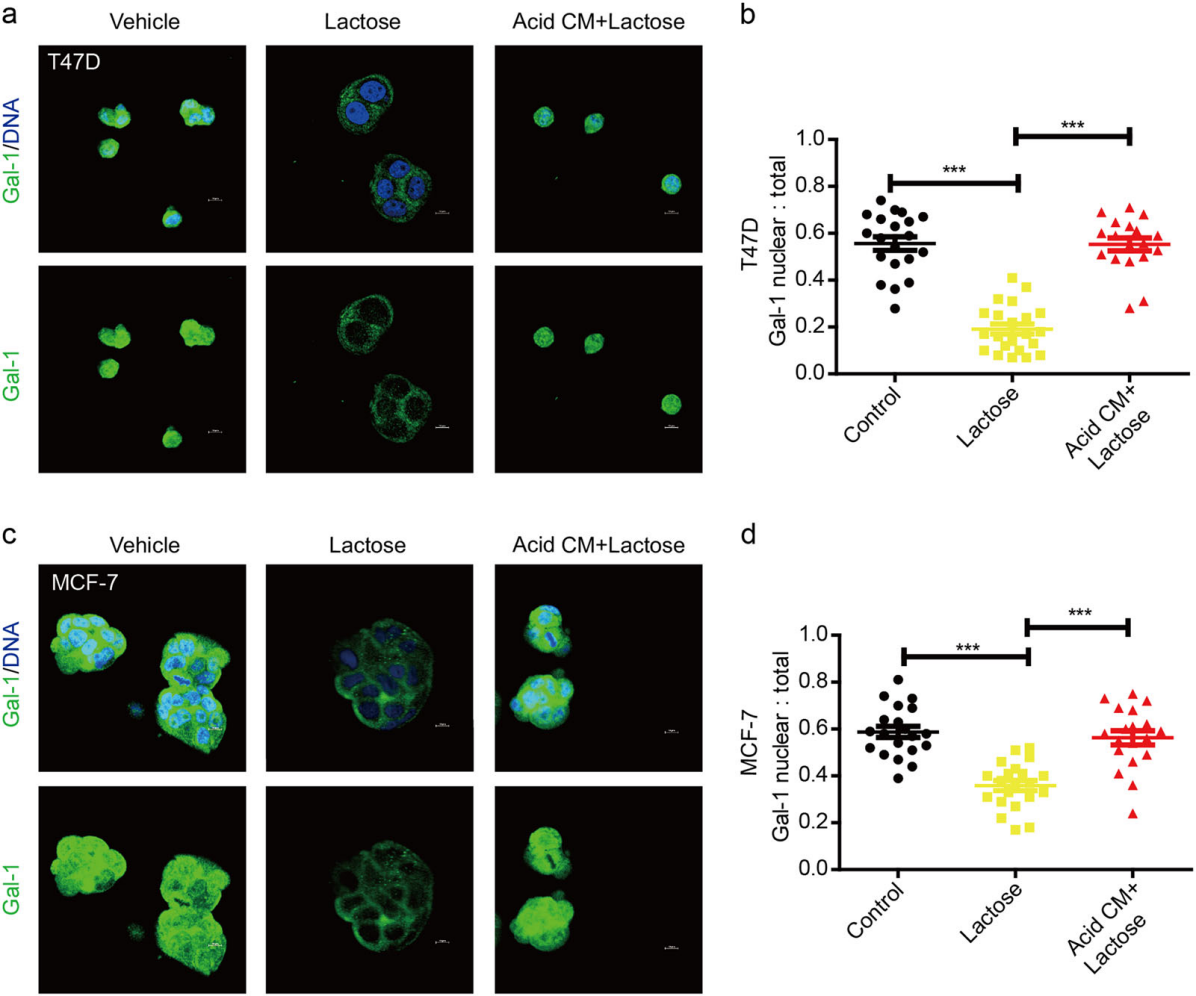
Extracellular lactose regulates the nuclear localization of Gal-1. **a** Confocal microscopy for the detection of Gal-1 expression in T47D cells embedded in 3D Matrigel-containing vehicle, soluble lactose, or conditional medium (CM) + lactose. Nuclear staining with DAPI is shown. Scale bar = 10 μm. **b** Quantification of nuclear:total Gal-1 ratio in T47D cells shown in **a**. **c** Confocal microscopy for the detection of Gal-1 expression in MCF-7 cells embedded in 3D Matrigel-containing vehicle, soluble lactose or conditional medium (CM) + lactose. Nuclear staining with DAPI is shown. Scale bar = 10 μm. **d** Quantification of nuclear:total Gal-1 ratio in MCF-7 cells shown in **c**. **b,d** The data are shown as the mean ± s.e.m. ***P < 0.001. **b,d** ANOVA with Dunnett’s *t*-test

Even though Gal-1 lacks recognizable secretory signal sequences, it is well known that intercellular Gal-1 can be secreted by cells through an unconventional mechanism associated with its glycan-binding activity^18^. To investigate whether the glycan-binding activity of Gal-1 regulates its subcellular localization, we re-engineered the microenvironment of breast cancer cells in vitro, by adding lactose to the cellular medium to mimic extracellular matrix glycan. The results showed that the nuclear localization of Gal-1 was lower in the T47D cells embedded in soluble lactose-containing 3D Matrigel than in cells embedded in control 3D Matrigel (Fig. 7a–b). In addition, it has been reported that LacNAc epitopes can be masked by sialic acid, resulting in the negative regulation of Gal-1 binding to lactose^29^. Furthermore, we found that the concentration of sialic acid was significantly higher in acid-conditional medium (CM), which was collected from breast cancer cells than that from the control medium (Supplementary Figure 6b–c). To mimic the acidic extracellular microenvironment in breast cancer tissues, the acidic CM from breast cancer cells was added to 3D Matrigel together with lactose. The results showed that the nuclear localization of Gal-1 was higher in the T47D cells embedded in CM + lactose-containing 3D Matrigel than that in cells embedded in soluble lactose-containing 3D Matrigel (Fig. 7a–b). A similar phenomenon was also observed using MCF-7 cells embedded in 3D Matrigel (Fig. 7c–d). The results suggested that the acidic extracellular microenvironment in breast cancer tissues might result in the accumulation of Gal-1 in the nucleus.

## Discussion

Nuclear FOXP3 is expressed in most of the normal breast epithelial cells, but lost in 70%–80% of breast cancer cells in human breast cancer samples^5^. In fact, the ability of FOXP3 to localize in the nucleus is a pre-requisite for its function as a transcription factor^6,30^. Our previous study showed that the reduction in the expression of nuclear FOXP3 protein was significantly associated with tumor progression in breast cancer patients^31^. Therefore, considering that somatic mutations of *FOXP3* are common in human breast cancer tissues, it is usually thought that the cytoplasmic localization of FOXP3, resulting from somatic mutation, is associated with the loss of its tumor-suppressive function^11,32^. However, in our previous study and the present one^31^, the expression of nuclear FOXP3 via immunohistochemistry was observed in a substantial number of breast cancer samples. Moreover, by analyzing single-cell RNA-sequencing data from primary breast cancer cells, we found that the full-length FOXP3 transcript was present in 54.5% of breast cancer samples. Therefore, the idea that cytoplasmic localization induces the loss of the tumor-suppressive property of FOXP3 is not sufficient to explain why the tumor-suppressive function of FOXP3 is lost in wild-type FOXP3-positive tumors.

It has been reported that protein–protein interaction could abrogate the tumor-suppressive function of BRCA1^33^, implying that the interaction of FOXP3 with some proteins may result in the loss of its tumor-suppressive properties in breast cancer. Using co-IP and FRET analysis, we found that the interaction of FOXP3 with Gal-1 occurred mainly in the nucleus of breast cancer cells. Furthermore, our results showed that the FKH domain of FOXP3 was essential for its interaction with Gal-1. The FKH domain is very important for the function of FOXP3. The absence of this region results in a failure of this protein to interact with DNA and thus act as a transcription factor^34^. Therefore, the binding of Gal-1 “masks” the FKH domain of FOXP3, which can result in the dysregulation of many oncogenes. Moreover, by ChIP-seq assay, we found that the differentially FOXP3-bound genes are involved in tumor development and metastasis^35–38^. The top 20 signal pathways enriched for FOXP3-bound genes mainly included cancer-related pathways, and these were almost abolished by the co-transfection of Gal-1. The subsequent cellular functional experiments indicated that, the inhibitory effects of FOXP3 on the proliferation and metastasis of breast cancer cells in vitro were indeed abolished by the interaction of FOXP3 and Gal-1.

As mentioned above, many studies have focused on the function of extracellular Gal-1, while the function of intercellular Gal-1 remains to be elucidated. To assess the possibility of interaction between FOXP3 and Gal-1 in vivo, we examined the expression of Gal-1 in 53 pairs of nuclear FOXP3-positive breast cancer tissue and adjacent normal tissue samples. The results showed that the abundance of nuclear Gal-1 was significantly higher in breast cancer tissues than in the adjacent normal tissues. These findings indicate that the tumor-suppressive property of FOXP3 is more likely to be negated due to the higher abundance of nuclear Gal-1 in breast cancer tissues. However, due to the lack of appropriate animal models, it has several limitations that must be acknowledged. How the blockade of Gal-1-FOXP3 interaction affects tumor progression in wild-type FOXP3-positive breast cancer animal models needs further study.

The results of the analysis of clinical specimens raise yet another key question: What factors promote the nuclear localization of Gal-1 in breast cancer tissues? Gal-1 is synthesized on cytosolic ribosomes and has a high affinity for LacNAc-enriched glycoconjugates^39^. Using 3D-culture systems, we observed that extracellular lactose induced a decrease in nuclear Gal-1. Due to elevated cell proliferation and glucose metabolism, the production and excretion of H^+^ are generally increased in cancers^40^. Combined with poor blood circulation, excess H^+^ can lead to an acidic extracellular microenvironment in malignant tumor tissues, compared to that in normal tissues^41,42^. It is reported that the malignant breast tissues have lower levels of LacNAc than the non-malignant tissue sections^29^. In our study, the acidic conditional medium of breast cancer cells was collected to mimic the acidic tumor microenvironment. Our results showed that during the 3D culture of breast cancer cells in this medium, lactose failed to redistribute Gal-1 to the extranuclear space. Some studies have also indicated that sialic acid can modify terminal LacNAc residues and mask the ligand of Gal-1, thereby interfering with the binding to Gal-1^43,44^. Interestingly, we found that the concentration of sialic acid was significantly higher in acid CM than that in control medium. Collectively, these observations suggest that in normal breast tissues, LacNAc epitopes in glycan can induce the extranuclear distribution of Gal-1, while in malignant breast tissues, LacNAc epitopes may be modified and masked, which results in the predominant localization of Gal-1 in the nucleus.

Our results demonstrate that Gal-1 is a novel FOXP3-interacting protein, and that nuclear Gal-1 can interact with FOXP3 and dampen its tumor-suppressive effects in breast cancer. Our findings not only identify a novel function of nuclear Gal-1 in breast cancer, but also suggest that blocking the Gal-1-FOXP3 interaction might be a promising treatment for wild-type FOXP3-positive breast cancer.

## Materials and Methods

### Cell culture

Human breast cancer cell lines MDA-MB-231, T47D, and MCF-7 were obtained from type culture collection of the Chinese Academy of Sciences (Shanghai, China). All cell lines were authenticated using STR profiling, and 100% of them matched the standard cell lines in the DSMZ data bank. All cells were negative for contamination with other human cells and mycoplasma.

The cells were cultured in an appropriate medium supplemented with 10% fetal bovine serum (FBS) (HyClone SH30068.03, South Logan, USA) and 100 µg/mL ampicillin/streptomycin.

### Antibodies and plasmids

The antibodies and dilutions used are as follows: FOXP3 (ab22510: immunoblotting, 1:500; immunofluorescence, 1:1000) from Abcam; FOXP3 (af3240: immunohistochemistry, 1:50) from R&D systems; Galectin-1 (ab138513: immunohistochemistry, 1:250) from Abcam; Galectin-1 (ab108389: immunoblotting, 1:1000; IP, 1:100; immunofluorescence, 1:250) from Abcam; GAPDH (CW0101: immunoblotting, 1:1000) from CWBIOTECH; c-MYC tag (10828-1-AP: immunoblotting, 1:500; IP, 1:100) from Proteintech; and Flag tag (14793: immunoblotting, 1:1000; IP, 1:50) from Cell Signaling Technology.

pcDNA3.1(-)-FOXP3, pcDNA3.1(-)-Galectin-1, PC-MYC-Galectin-1, pDsRed1-Gal-1, pEGFP-FOXP3, and pFLAG-FOXP3 plasmids were stored in our laboratory. The synthesized mutant Galectin-1 was digested with Kpn1 and Xho1, and cloned into the PC-MYC vector. The FOXP3-truncation mutants were digested with EcoRI/BamHI, and cloned into the pFLAG vector.

### Clinical specimens and immunohistochemistry

Patient tumors were staged and classified according to the American Joint Committee on Breast Cancer Staging and Classification criteria. In total, 165 pairs of breast cancer and adjacent normal samples were obtained from the Department of Pathology, The First Affiliated Hospital to The Fourth Military Medical University (FMMU, Shaanxi, China).

Immunohistochemistry was performed, as previously described^45^. Serial sections (4 μm) of paraffin-embedded samples were deparaffinized and rehydrated with an ethanol gradient. After the inactivation of endogenous peroxidase with 3% H_2_O_2_-methanol for 10 min, the sections were washed three times in PBS and blocked with goat serum for 20 min. Then, the sections were coated with primary antibodies, and incubated in a humid box at 4 °C overnight. After the addition of PowerVision^TM^ complex, tumor sections were incubated at 37 °C for 20 min, followed by DAB labeling to develop a brown color. PBS was used instead of antibodies, as a negative control. With respect to the expression of FOXP3, each specimen was classified as “Positive” or “Negative”. Sections positive in both nucleus and cytoplasm or only nucleus were considered “Positive.” Nuclear staining for Galectin-1 was quantified using the immunohistochemistry H-score: H score = ∑Pi × (*i* + 1), where *i* is the nuclear intensity score (range 0–4) and Pi is the percentage of stained tumor cells at each intensity (range 0%–100%). A score with a range of 100–500 was then produced, where 100 indicates that 100% of the tumor cells were negative, and 500 indicates that 100% of the tumor cells were strongly stained.

### Fluorescence resonance energy transfer analysis

Briefly, breast cancer cells were seeded in 6-well culture plates (at a density of 5 × 10^5^ cells/well), and co-infected with recombinant FOXP3-eGFP and recombinant Gal-1-dsRed. After 48 h, FRET microscopy (Nikon A1R, Japan) was performed to observe the expression of eGFP or dsRed fusion protein, while maintaining the cells in a humidified atmosphere with 5% CO_2_, 17% O_2_, and 78% N_2_ at 37 °C. Then, the software NIS-Elements was used to calculate FRET efficiency.

### Docking protocol

We docked the X-array structure of FOXP3 (PDB ID: 4WK8), which was defined here as a receptor, to the X-array structure of Gal-1 (PDB ID: 4Y24) using the ClusPro 2.0 software (a widely used tool for protein–protein docking)^46,47^. Based on the docked structure, we determined the interface residuals. The interface residues were defined as residues within 4.5 Å distance in the predicted complex.

### Western blot analysis

The cells were lysed on ice, according to the instructions. Protease Inhibitor Cocktail (MedChem Express, Monmouth Junction, USA) was used in cell lysates to increase protein stability. After SDS-PAGE, the proteins were transferred to PVDF membranes (0.22 µm, Invitrogen), using a Bio-Rad Semi-Dry Electrophoretic Transfer Cell. Western blot analyzes were performed, using corresponding specific antibodies, followed by HRP-conjugated IgG antibody. An enhanced chemiluminescence against HRP was used for the visualization of immunoreactive proteins.

### ChIP-Seq analysis

Briefly, 2 × 10^8^ breast cancer cells were collected from eight individual samples. The cells were fixed for 10 min at room temperature with 1% formaldehyde-containing medium and sonicated ten times for 5 s each. The sonicated chromatin samples were incubated at 4 °C overnight, with the anti-FOXP3 antibody. From each ChIP reaction mixture, 10% was kept as input DNA. Pre-rinsed protein A/G plus agarose beads (final concentration 75 ng/μl) were added to each ChIP reaction mixture and incubated for 60 min at room temperature. The beads were then incubated in 100 μl of elution buffer at 65 °C for 20 min to elute the immunoprecipitated DNA. ChIP-Seq libraries were prepared, using a Paired-End DNA Sample Prep kit (Illumina, PE-102-1001), and were run on HiSeq 4000 Sequencing Systems (Illumina). All sequencing data were mapped to the February 2009 human genome assembly (GRCh37/hg19), and peak calling was performed, using the Model-based Analysis of ChIP-Seq (MACS) version 2.1.1 (http://liulab.dfci.harvard.edu/MACS) with the default parameters to obtain the primary binding regions. For ChIP-Seq analysis, the background was subtracted, and the primary binding regions were filtered based on 5–50-fold enrichment and *q* value < 0.01.

### Quantitative real-time PCR

s, with RNAiso Plus (Takara, Dalian, China), and cDNA was synthesized with the PrimeScript RT Reagent Kit (Takara). Then, 2 ml of cDNA was used for real-time PCR reactions in a Prism 7500 real-time thermocycler (Applied BioSystems, FosterCity, CA, USA) with SYBR Green Ex Taq (Takara), according to the manufacturer’s instructions. The primer sequences are provided in Supplementary Table 5.

### xCELLigence Real-Time Cell Analysis (RTCA)

Experiments were carried out with the xCELLigence RTCA DP instrument (Roche Diagnostics GmbH, Mannheim, Germany), placed in a humidified incubator at 37 °C with 5% CO_2_. Modified 16-well plates (E-plate, Roche Diagnostics GmbH, Mannheim, Germany) were used in cell proliferation experiments^48^. First, 50 μL of cell-free growth medium was added to the wells. After measuring the background impedance for each well, 100 μL of cell suspension was seeded into each well. The impedance value of each well (cell index value) was automatically monitored using the xCELLigence system every 15 min for 72 h. Each group was analyzed in quadruplicate.

### Invasion assays

Breast cancer cells (1 × 10^5^) were plated in transwell chambers coated with 100 μl of Matrigel. Then, the chambers were inserted into a 24-well plate and incubated in corresponding medium with 10% FBS for 24 h before examination. The cells remaining on the upper surface of the membrane were removed, while the cells that had migrated to the lower surface were fixed with 95% ethanol and stained in 4 g/L crystal violet solution. Three fields of view were finally selected to count the number of invasive cells under a microscope, and the relative cell counts were averaged.

### Immunofluorescence analysis

To determine the subcellular localization of Gal-1, 2 × 10^5^ breast cancer cells were embedded in Matrigel (containing vehicle, soluble lactose or acidic conditional medium + soluble lactose) and incubated at 37 °C overnight. Then, the cells were washed twice with cold PBS, fixed with 4% paraformaldehyde for 20 min, and permeabilized with 0.2% Triton X-100 for 30 min at room temperature. The cells were incubated overnight at 4 °C with corresponding antibodies, after being blocked for 30 min with goat serum. Then, the cells were incubated for 2 h with secondary FITC-conjugated or Cy3-conjugated antibodies. Images were captured using a confocal microscope (FluoView^TM^ FV1000, Olympus).

### Sialic acid assay

After incubation for 60 h, the culture medium of breast cancer cells was collected as the acidic conditional medium. This assay was performed with Sialic Acid (NANA) Assay Kit (abcam, ab83375). The concentration of sialic acid was detected in each medium, according to the manufacturer’s instructions.

### Statistical analysis

The data were represented as the mean ± s.e.m from at least three independent experiments. Differences were considered statistically significant when *P* < 0.05. Statistical analyzes were performed using SPSS software (SPSS 16.0, Chicago). All statistical tests were two-sided.

## Electronic supplementary material

supplemental figures, tables(DOCX 21417 kb)
