# Supplementary material for: Nuclear galectin-1-FOXP3 interaction dampens the tumor-suppressive properties of FOXP3 in breast cancer
Source: Cell Death Dis. 2018 Mar 16;9(4):416. doi: 10.1038/s41419-018-0448-6 (PMC5856744; doi:10.1038/s41419-018-0448-6)
Supplement: Supplementary file 1 — supplemental figures, tables(DOCX 21417 kb) [file 41419_2018_448_MOESM1_ESM.docx]

**Nuclear galectin-1-FOXP3 interaction dampens the tumor-suppressive properties of FOXP3 in breast cancer**

**Supplementary files: (Figures, and Tables)**

**Supplementary Figure 1**

**
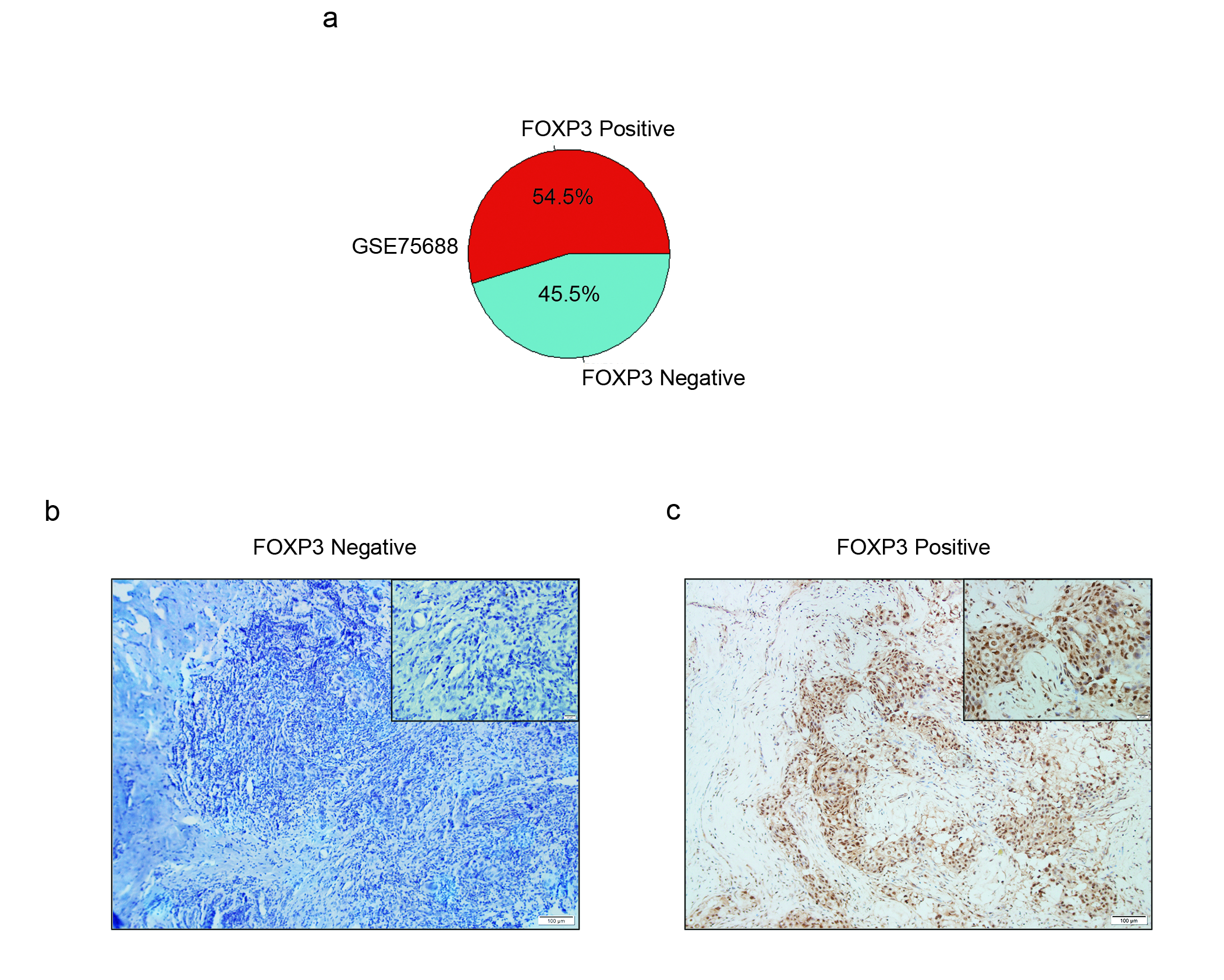
**

**Supplementary Figure 1: The expression of FOXP3 is detected in breast cancer tissues.** (**a**) The expression level of the full-length FOXP3 transcript was analyzed in breast cancer cells in the GSE75688 dataset. (**b-c**) Representative immunohistochemical staining images for FOXP3 in primary breast tumors. Scale bars represent 100 μm (10×) and 20 μm (40×).

**Supplementary Figure 2**

**
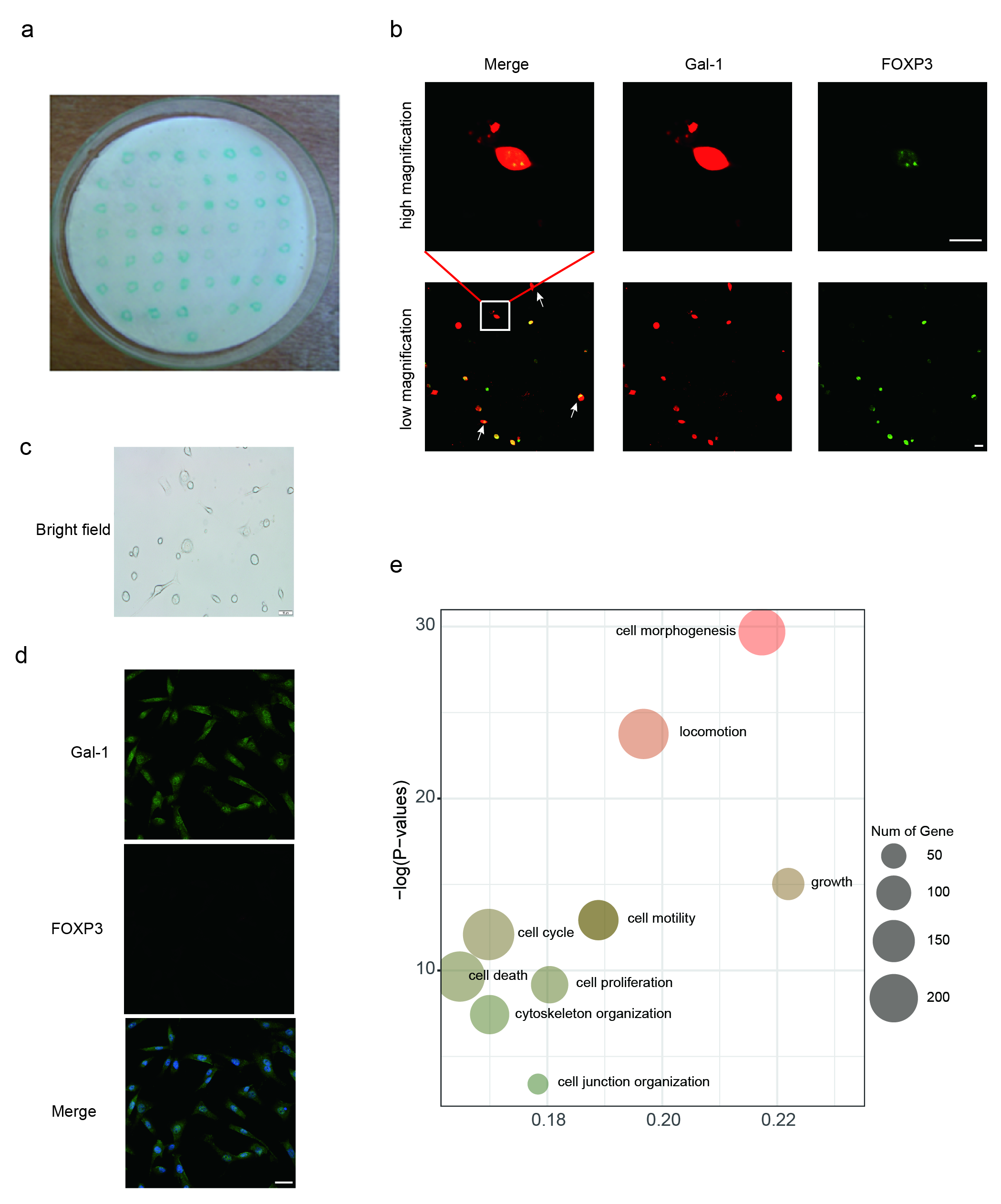
**

**Supplementary Figure 2:** (**a**) Identification of Gal-1 as a potential FOXP3-binding partner using yeast two-hybrid screening. (**b-c**) The FOXP3-eGFP and Gal-1-dsRed fusion proteins were constructed and co-transfected into MDA-MB-231 cells. (**b**) Confocal assay for FOXP3-eGFP and Gal-1-dsRed localization in MAD-MB-231 cells. The white arrow indicates nuclear colocalization of these proteins. Scale bar represents 20 μm (high) or 30 μm (low). (**c**) Representative picture of these cells in bright field. Scale bar represents 50 μm. (**d**) Confocal assay for native FOXP3 and Gal-1 expression in MAD-MB-231 cells. Scale bar represents 30 μm. (**e**) GO analysis of the differentially FOXP3-bound genes.

.

**Supplementary Figure 3**


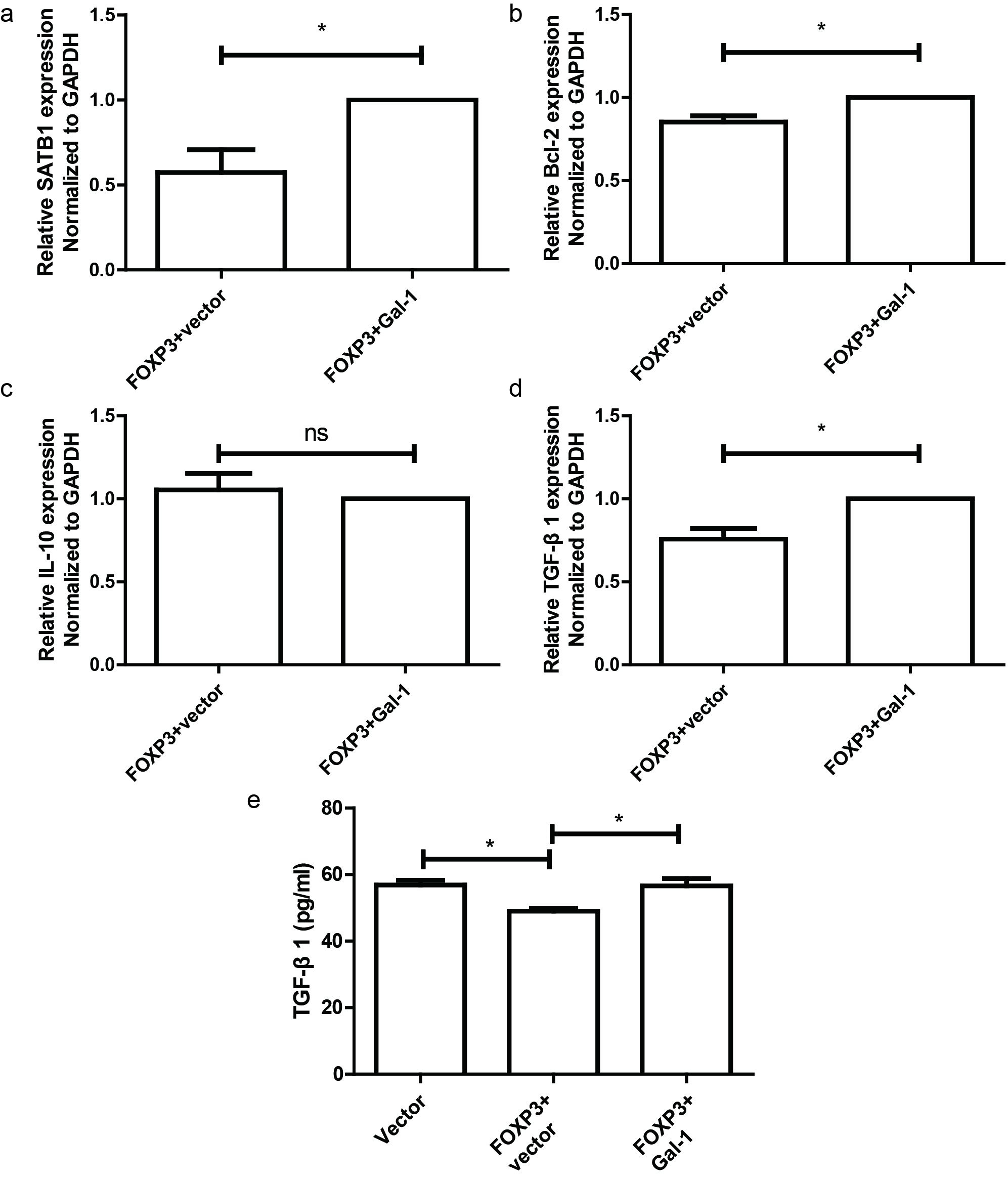


**Supplementary Figure 3:** (**a**) Real-time PCR detecting the transcription levels of SATB1 in breast cancer cells. The results were normalized to GAPDH (n=3) (**b**) Real-time PCR detecting the transcription levels of Bcl-2 in breast cancer cells. The results were normalized to GAPDH (n=3) (**c**) Real-time PCR detecting the transcription levels of IL-10 in breast cancer cells. The results were normalized to GAPDH (n=3) (**d**) Real-time PCR detecting the transcription levels of TGF-β1 in breast cancer cells. The results were normalized to GAPDH (n=3) (**e**) After the knockdown of endogenous Gal-1, FOXP3-MDA-MB-231cells or MDA-MB-231 cells were transfected with Gal-1 or empty vector. The TGF-β1 level in supernatant was detected by ELISA assay. (**a, b, c, d, e**) The data are shown as the mean ± s.e.m. ns *P*>0.05, **P*<0.05, (**a, b, c, d**) Student *t* test. (**e**) ANOVA with Dunnett *t* test.

**Supplementary Figure 4**

**
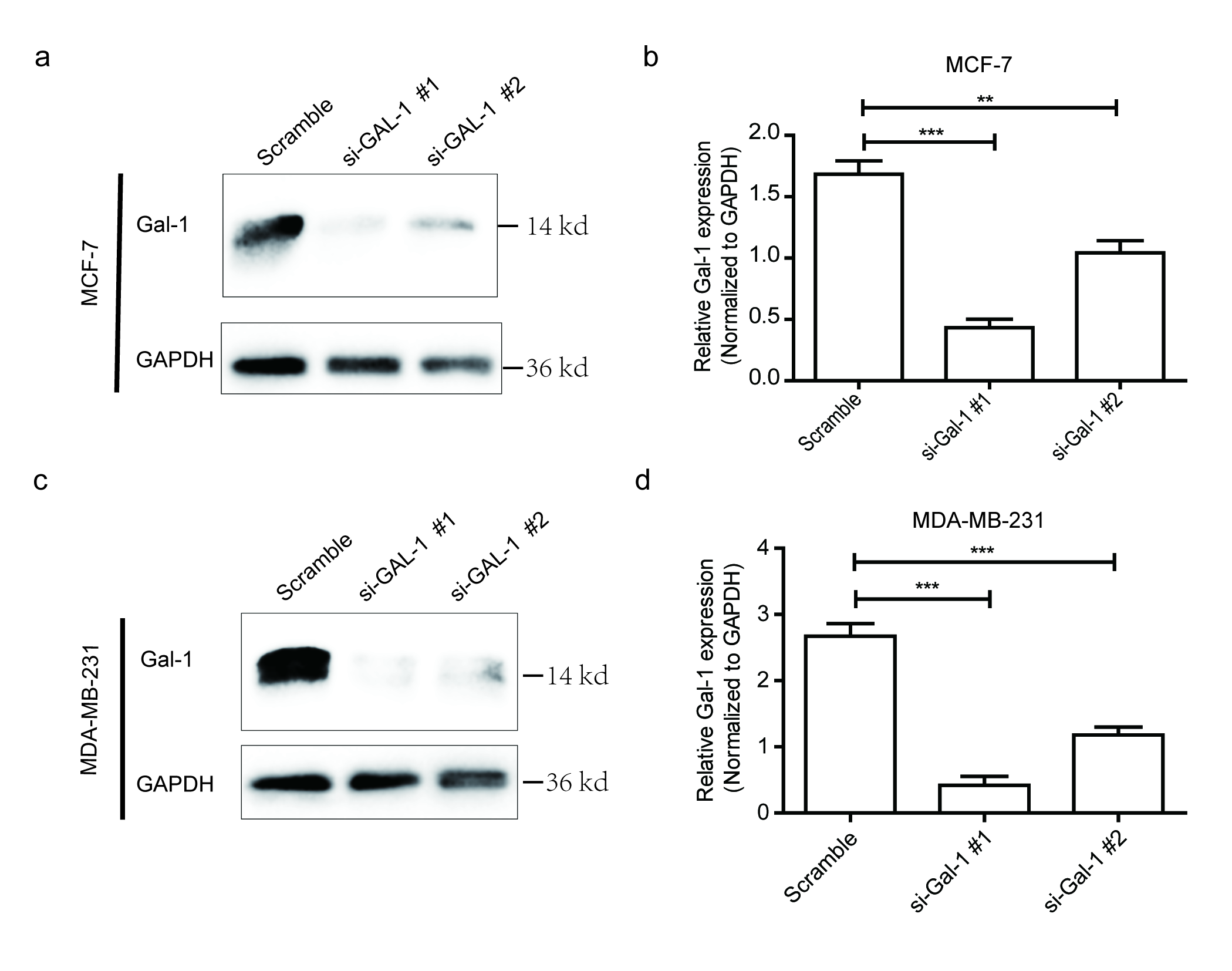
**

**Supplementary Figure 4:** (**a**) Western blot analysis for the evaluation of interference efficiency of Gal-1-specific siRNAs in MCF-7 cells. (**b**) Quantification of Gal-1 expression normalized to GAPDH in MCF-7 cells (n=3). (**c**) Western blot analysis for the evaluation of interference efficiency of Gal-1-specific siRNAs in MDA-MB-231 cells. (**d**) Quantification of Gal-1 expression normalized to GAPDH in MDA-MB-231 cells (n=3). (**b, d**) The data are shown as the mean ± s.e.m. ***P*<0.01, ****P*<0.001 (**b, d**) ANOVA with Dunnett *t* test.

**Supplementary Figure 5**


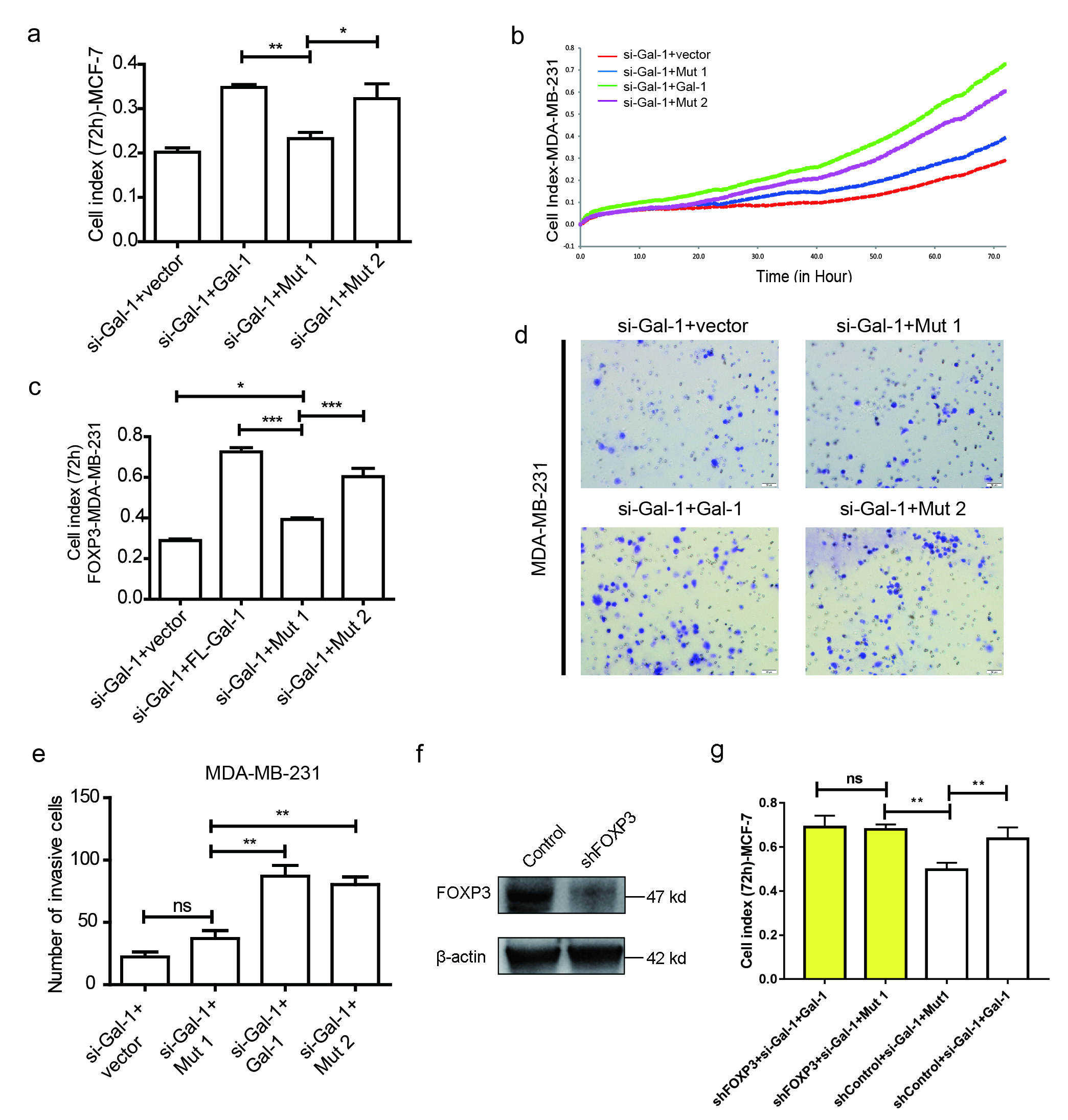


**Supplementary Figure 5**: (**a**) The cellular index of MCF-7 cells (related to Figure 5a) after 72 h. (**b)** siRNAs specifically targeting Gal-1 were transfected into FOXP3-overexpressing MDA-MB-231 cells to knockdown endogenous Gal-1, and then these cells were transfected with vectors expressing Mut1, Mut2, or Gal-1 or empty vector and subjected to xCELLigence RTCA. (**c**) The cellular index of MDA-MB-231 cells in b (after 72 h). (**d**) A transwell assay was performed to determine the invasive capability of si-Gal-1-transduced and FOXP3 overexpressing MDA-MB-231 cells transfected with vectors expressing Mut1, Mut2, or Gal-1 or empty vector. Scale bar represents 50 μm. (**e**) Quantification of invasive cells from d (n=3). (**f**) Western blot analysis for the evaluation of interference efficiency of FOXP3-specific shRNA in MCF-7 cells. (**g**) The cellular index of MCF-7 cells (related to Figure 5f) after 72 h. (**a, c, e, g**) The data are shown as the mean ± s.e.m. ns *P*>0.05, **P*<0.05, ***P*<0.01, ****P*<0.001. (**a, c, e**) ANOVA with Dunnett’s *t* test. (**g**) ANOVA with Tukey *t* test.

**Supplementary Figure 6**


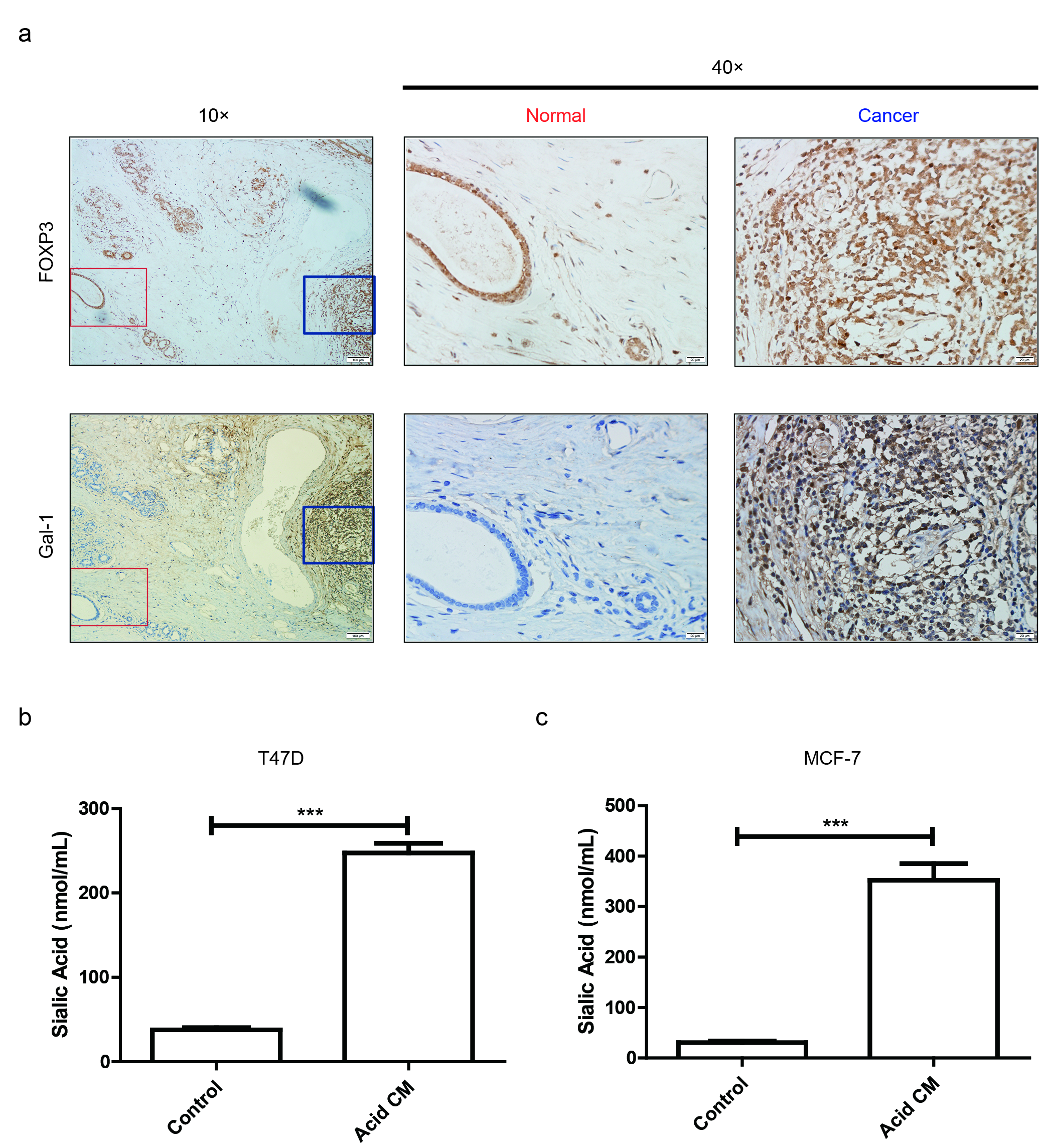


**Supplementary Figure 6**: (**a**) Representative immunohistochemical staining for FOXP3 and Gal-1 in normal tissues (red) and primary tumor tissues (blue) from breast cancer patients. Scale bars represent 100 μm (10×) and 20 μm (40×). (**b**) The concentration of sialic acid was detected in control medium or acid CM from T47D cells. (**c**) The concentration of sialic acid was detected in control medium or acid CM from MCF-7 cells. (**b，c**) The data are shown as the mean ± s.e.m. ****P*<0.001 (**b，c**) Student *t* test.

**Supplementary Table 1: Association between the expression of FOXP3 and breast carcinoma characteristics**

| **Variables** | | **Total** | **FOXP3 expression** | | | ***P*-value** | |
| --- | --- | --- | --- | --- | --- | --- | --- |
|  |  |  | **+** | **-** |  | | |
|  | **N=165** | | **N=53** | **N=112** |  | | |
| Age(year) | |  |  |  |  | | |
| ≤ 50 | | 99 | 31 (31/99, 31.31%) | 68 (68/99, 68.69%) | | | 0.7854 |
| ＞50 | | 66 | 22 (22/66, 33.33%) | 44 (44/66, 66.67%) | | |  |
| Total | | 165 | 53 | 112 | | |  |
| Clinical stages | |  |  |  | | |  |
| AJCC Ⅰ | | 38 | 13 (13/38, 34.21%) | 25 (25/38, 65.79%) | | | **<0.0001** |
| AJCC Ⅱ | | 65 | 21 (21/65, 32.31%) | 44 (44/65, 67.69%) | | |  |
| AJCC Ⅲ | | 62 | 19 (19/62, 30.65%) | 43 (43/62, 69.35%) | | |  |
| Total | | 165 | 53 | 112 | | |  |
| ER status | |  |  |  | | |  |
| ER+ | | 118 | 34 (34/118, 28.81%) | 84 (84/118, 71.19%) | | | 0.1494 |
| ER- | | 47 | 19 (19/47, 40.43%) | 28 (28/47, 59.57%) | | |  |
| Total | | 165 | 53 | 112 | | |  |
| PR status | |  |  |  | | |  |
| PR+ | | 108 | 33 (33/108, 30.56%) | 75 (75/108, 69.44%) | | | 0.5533 |
| PR- | | 57 | 20 (20/57, 35.09%) | 37 (37/57, 64.91%) | | |  |
| Total | | 165 | 53 | 112 | | |  |
|  | |  |  |  | | |  |

Statistical analysis of “Age, PR status, ER status” was performed with the chi-square test; Statistical analysis of “Clinical stages” was performed with the Wilcoxon rank sum test.

**Supplementary Table 2: Proteins that may interact with FOXP3 in Yeast Two-hybrid assay**

| **Number** | **Protein** |
| --- | --- |
| **1** | Lymphotoxin B |
| **2** | Ubiquitously Expressed Transcript |
| **3** | Actin |
| **4** | Galectin-1 |
| **5** | Ficolin 1 |
| **6** | [Carboxy-peptidase](javascript:void(0);) |
| **7** | Homeodomain Interacting Protein Kinase 1 |
| **8** | Eukaryotic Initiation Factor 3 |

**Supplementary Table 3: The sequence of Gal-1 siRNAs**

| **ID** | **sense（5'-3'）** | **antisense（5'-3'）** |
| --- | --- | --- |
| LGALS1-1 | 5' GAUGGAUACGAAUUCAAGUUC 3' | 5' ACUUGAAUUCGUAUCCAUCUG 3' |
| LGALS1-2 | 5' GAUGGAUACGAAUUcaagUUc 3' | 5' acUUgaAUUCGUAUCCAUCUG 3' |
| Negative control | 5' UUCUCCGAACGUGUCACGUTT 3' | 5' ACGUGACACGUUCGGAGAATT 3' |

**Supplementary Table 4: Association between the expression of nuclear Gal-1 and breast carcinoma characteristics**

| **Variables** | **Total** | | **Nuclear Gal-1 expression** | | ***P*-value** | |
| --- | --- | --- | --- | --- | --- | --- |
|  |  |  | **High**  **(H-score>** **median)** | **Low**  **(H-score≤median) mmmmedianmedian H-score < median** | |  |
|  | | **N=53** | **N=26** | **N=27** | |  |
| Age(year) |  | |  |  | | 0.501 |
| ≤ 50 | 31 | | 14 (14/31, 45.16%) | 17 (17/31, 54.84%) | |  |
| ＞50 | 22 | | 12 (12/22, 54.55%) | 10 (10/22, 45.45%) | |  |
| Total | 53 | | 26 | 27 | |  |
| Clinical stages |  | |  |  | | 0.236 |
| AJCC Ⅰ | 13 | | 7 (7/13, 53.85%) | 6 (6/13, 46.15%) | |  |
| AJCC Ⅱ | 21 | | 9 (9/21, 42.86%) | 12 (12/21, 57.14%) | |  |
| AJCC Ⅲ | 19 | | 10 (10/19, 52.63%) | 9 (9/19, 47.37%) | |  |
| Total | 53 | | 26 | 27 | |  |
| ER status |  | |  |  | | 0.125 |
| ER+ | 34 | | 14 (14/34, 41.18%) | 20 (20/34, 58.82%) | |  |
| ER- | 19 | | 12 (12/19, 63.16%) | 7 (7/19, 36.84%) | |  |
| Total | 53 | | 26 | 27 | |  |
| PR status |  | |  |  | | 0.500 |
| PR+ | 33 | | 15 (15/33, 45.45%) | 18 (18/33, 54.55%) | |  |
| PR- | 20 | | 11 (11/20, 55.00%) | 9 (9/20, 45.00%) | |  |
| Total | 53 | | 26 | 27 | |  |

Statistical analysis of “Age, PR status, ER status” was performed with the chi-square test; Statistical analysis of “Clinical stages” was performed with the Wilcoxon rank sum test.

**Supplementary Table 5: The sequence of primer sets for real-time PCR assay**

| ID | Forward | Reverse |
| --- | --- | --- |
| SATB1 | TTGAACGAGGCAACTCAGGG | CATCAGATGGCCCGAGTGTT |
| Bcl-2 | GTGGAGGAGCTCTTCAGGGAC | CCGGTTCAGGTACTCAGTCATCC |
| IL-10 | CGAGATGCCTTCAGCAGAGT | CGCCTTGATGTCTGGGTCTT |
| TGF-β1 | TGGTGGAAACCCACAACGAA | GAGCAACACGGGTTCAggTA |
| GAPDH | GCTCGTCGTCGACAACGGCTC | CAAACATGATCTGGGTCATCTTCTC |
